# The Association of a Genetic Variant in* SCAF8-CNKSR3* with Diabetic Kidney Disease and Diabetic Retinopathy in a Chinese Population

**DOI:** 10.1155/2017/6542689

**Published:** 2017-03-19

**Authors:** Li Jin, Tao Wang, Song Jiang, Miao Chen, Rong Zhang, Cheng Hu, Weiping Jia, Zhihong Liu

**Affiliations:** ^1^National Clinical Research Center of Kidney Diseases, Jinling Hospital, Nanjing University School of Medicine, Nanjing 210016, China; ^2^Shanghai Diabetes Institute, Shanghai Key Laboratory of Diabetes Mellitus, Shanghai Clinical Center for Diabetes, Shanghai Jiao Tong University Affiliated Sixth People's Hospital, Shanghai 200233, China

## Abstract

*Background*. Genome-wide association studies found rs955333 located in 6q25.2 was associated with diabetic kidney disease in multiple ethnic populations, including European Americans, African Americans, and Mexican Americans. We aimed to investigate the association between the variant rs955333 in* SCAF8-CNKSR3* and DKD susceptibility in Chinese type 2 diabetes patients.* Methods*. The variant rs955333 was genotyped in 1884 Chinese type 2 diabetes patients. Associations of the variant rs955333 with DKD and DR susceptibility and related quantitative traits were evaluated.* Results*. The variant rs955333 was not associated with DKD in our samples, while subjects with genotype GG were associated with DR (*P* = 0.047, OR = 0.5525 [0.308,0.9911]), and it also showed association with microalbuminuria (*P* = 0.024, beta = −0.1812 [−0.339, −0.024]).* Conclusion*. Our data suggests the variant rs955333 was not associated with DKD but showed association with diabetic retinopathy in Chinese type 2 diabetes patients.

## 1. Introduction

Diabetic kidney disease is a leading cause of end-stage kidney disease (ESKD) globally and continues to grow over decades [1]. A recent study shows that chronic kidney disease accompanied diabetes has become more widespread than primary glomerulonephritis in China [2]. Intensification of glycemic and blood pressure and lipid control has not markedly reduced the incidence of DKD [3]. There might be any other factors which play an important role in occurrence and development of the disease [4]. The evidence including aggregation in families, variable prevalence rates between different race, and the highly heritable trait in clinic and histology indicates that genetic factors play an important role in the pathogenesis of DKD [5–8]. Genome-wide association studies have identified that several loci are associated with DKD in different population [9–14].

A genetic variant rs955333 within* SCAF8-CNKSR3* has been identified to be associated with DKD and reach genome-wide significance in multiple ethnic populations, including European Americans, African Americans, and Mexican Americans [15]. This Single Nucleotide Polymorphism (SNP) is located in a region between the SR-like carboxyl-terminal domain associated factor 8 gene* (SCAF8)* and the connector enhancer of KSR family of scaffold proteins gene* (CNKSR3)*, which may suggest that this SNP may regulate transcription of genes in the region [15].* CNKSR3* is an aldosterone receptor target gene and it mainly regulates sodium absorption in the distal nephron to maintain plasma volume and blood pressure [16].* SCAF8* is RNA maturation factor and plays a role in mRNA processing [15].

Although this SNP shows strong relationship with DKD in FIND's study [15], there is no data about its association with DKD in Chinese population and relevant quantitative trait. Therefore, we performed the present study, aimed to test if rs955333 played a role in genetic susceptibility of DKD in Chinese population.

## 2. Methods

### 2.1. Ethical Approval

According to Helsinki Declaration II, ethical approval was granted by the institutional review board of Shanghai Jiao Tong University Affiliated Sixth People's Hospital. Oral and written informed consent were obtained from each participant.

### 2.2. Participants

In this study, we recruited 1884 unrelated Chinese Han subjects from the Shanghai Diabetes Institute Inpatient Database of Shanghai Jiao Tong University Affiliated Sixth People's Hospital. All participants were T2DM patient who were eastern Chinese Han ancestry and meet the 1999 WHO criteria (fasting plasma glucose ≥ 7.0 mmol/L and/or 2 h plasma glucose ≥ 11.1 mmol/L). Type 1 diabetes and mitochondrial diabetes were excluded by clinical, immunological, and genetic criteria, and patients with an estimated glomerular filtration rate albuminuria ≥ 30 mg/24 h or (eGFR) < 90 mL/min per 1.73 m^2^ or ACR (albumin-to-creatinine ratio) ≥ 30 *μ*g/mg were diagnosed as DKD. Of these patients, 508 were diagnosed with diabetic kidney disease and 1376 had diabetes for longer than 5 years but without diabetic kidney disease and were selected as cases and controls, respectively, 545 were diagnosed with diabetic retinopathy, and 1120 subjects who had diabetes for longer than 5 years but without diabetic kidney disease were chosen as control.

### 2.3. Clinical Measurement

The history of every patient was recorded in detail, as well as the anthropometric and biochemical data. The patients' height (/m) and weight (/kg) were measured, and their body mass index (BMI) was calculated as weight/height^2^. Blood pressure was also measured by standard measurements. HbA1c levels were surveyed by high performance liquid chromatography (HPLC) by a Bio-Rad Variant II Haemoglobin Testing System (Bio-Rad Laboratories, Hercules, CA, USA). The albuminuria level was measured with scatter turbidimetry by BN II System (Siemens Healthcare Diagnostics Products GmbH, Marburg, Germany), the sample came from 24 h urine collection, the measurement was repeated in three different days, and the mean values of these measurements were used for further analysis. The eGFR was calculated by a modification of the diet in renal disease (MDRD) study equation specially designed for a Chinese population [17]. Fundus photography of all subjects was performed with a 45° 6.3 megapixel digital nonmydriatic camera (Canon CR6-45NM; Lake Success, NY, USA).

### 2.4. SNP Selection, Genotyping, and Quality Control

The SNP reported by Family Investigation of Nephropathy and Diabetes (FIND), which reaches genome-wide significance, were genotyped. Genotyping was performed by primer extension of multiplex products with detection by matrix-assisted laser desorption ionisation-time of flight mass spectroscopy by a MassARRAY Compact Analyzer (Sequenom, San Diego, CA, USA). Approximately, 1884 individuals and SNP rs955333 were reserved for further analysis after the quality control.

### 2.5. Statistical Analysis

The allelic frequencies were compared between patients with or without DKD using a *χ*^2^ test in PLINK (v1.07; http://pngu.mgh.harvard.edu/~purcell/plink) [18] and ORs with 95% CIs are presented. Genotype distributions between patients with or without DKD were compared using logistic regression under additive and recessive models with adjustment of confounding factors. Quantitative traits related to diabetic microvascular disease were preformed using multiple linear regression under additive and recessive models with adjustment of confounding factors. The statistical analyses were performed using SAS 9.3 (SAS institute, Cary, NC, USA) unless specified otherwise. A two-tailed *P* value < 0.05 was considered statistically significant. On the basis of an estimated effect size of genetic loci for DKD (~0.73), our samples had >95% power to detect SNP with minor allele frequency of 0.2 at a level of significance of 0.05 by Quanto [19].

## 3. Results

The SNP rs955333 were successfully genotyped in 1884 individuals in the present study, without the basis of deviations from Hardy-Weinberg equilibrium (*P* = 0.77 in all subjects). We analyzed the association between the SNP and DKD and DR and related quantitative trait. The clinical characteristics of control and case of DKD or DR were shown in Tables 1 and 2, respectively. The results showed that there are significant differences between DKD cases and controls in gender, age, systolic blood pressure, diastolic blood pressure, AER, eGFR, uric acid, creatinine, blood urea nitrogen, total cholesterol, and total triglycerides (*P* < 0.05). Similarly, age, duration of diabetes, systolic blood pressure, and AER are also significantly different between DR cases and controls (*P* < 0.05).

**Figure 1:**
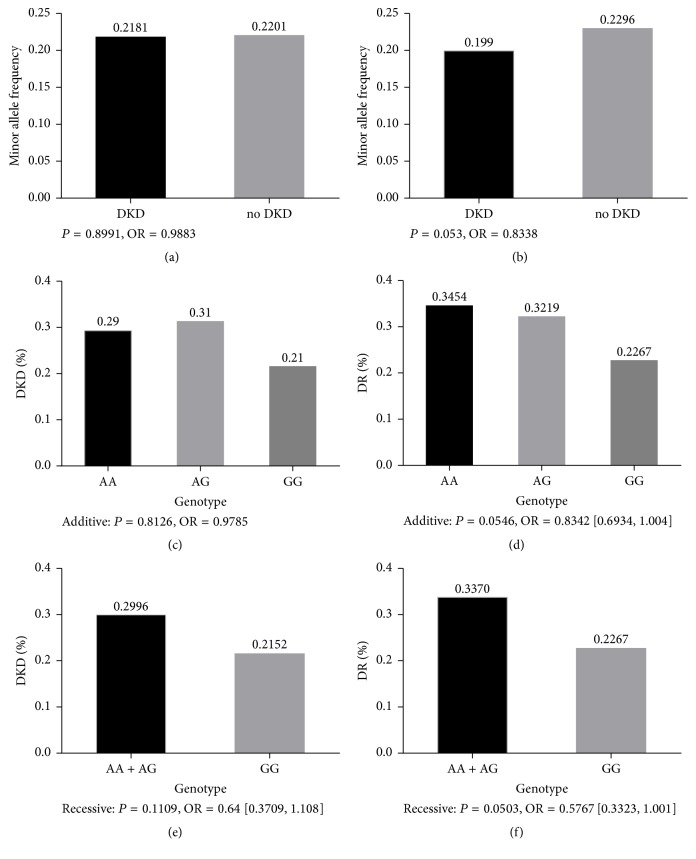
Allele frequency and prevalence rate in DKD and DR case-control study. (a) and (b) represent the association analysis in two case-control groups. The histograms represent the frequency of rs955333[G]. (c) and (d) represent the difference of prevalence rate in different genotypes on additive model. The histograms represent the prevalence rate. (e) and (f) represent the difference of prevalence rate in different genotypes on recessive model. The histograms represent the prevalence rate.

The allele frequency of the variant showed no difference between DKD group and no-DKD group (*P* = 0.8991) (Figure 1(a)), while it showed some level of difference between diabetic retinopathy group and no-diabetic retinopathy group (*P* = 0.053) (Figure 1(b)).

Then we tested the association between the SNP and susceptibility of DKD under additive or recessive model, respectively. It showed that genotype GG was also lack of association with DKD under additive model (*P* = 0.7003) and recessive model (*P* = 0.1109, OR = 0.64  [0.3709,1.108]) (Figure 1(c)). After that, we used logistic regression adjusting confounding factors including age, gender, BMI, duration, Hba1c, systolic blood pressure, diastolic blood pressure, total cholesterol, and total triglycerides to analyze the association between this rs955333 and DKD. Results suggested no association.

We also tested the association between the SNP and susceptibility of DR under additive or recessive model, respectively. It showed that rs955333 might be associated with DR both on the additive model (*P* = 0.0546, OR = 0.8342  [0.6934,1.004]) and on the recessive model (*P* = 0.0503, OR = 0.5767  [0.3323,1.001]) (Figure 1(d)). After adjusting confounding factors including age, gender, BMI, duration, Hba1c, systolic blood pressure, diastolic blood pressure, total cholesterol, and total triglycerides, the result showed that subjects with genotype GG were associated with DR (*P* = 0.047, OR = 0.5525  [0.308,0.9911]).

**Figure 2:**
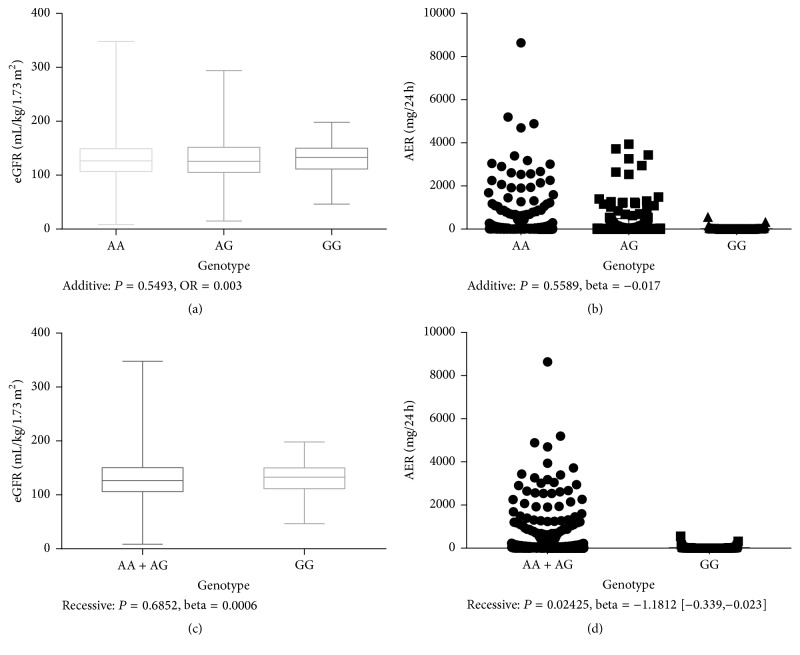
The difference of eGFR and AER in different genotypes. Box plot (a) shows the association of rs955333 with eGFR on additive model. Scatter plot (b) shows the association of rs955333 with AER on additive model. Box plot (c) shows the association of rs955333 with eGFR on recessive model. Scatter plot (d) shows the association of rs955333 with AER on recessive model. *P* values and beta values were determined by multiple linear regression adjusting for age, gender, BMI, duration, Hba1c, DP, SP, TC, and TG.

As for the association of the variant rs955333 with eGFR and 24-hour urinary albumin excretion rate (AER) by multiple linear regression analysis adjusting confounding factors including age, gender, BMI, duration, Hba1c, systolic blood pressure, diastolic blood pressure, total cholesterol, and total triglycerides, it showed association with AER (*P* = 0.024, beta = −0.1812  [−0.339, −0.024]) (Figure 2).

## 4. Discussion

Our study aimed to test the genetic association between SNP rs955333 and diabetic kidney disease in Chinese population. The DKD related SNP which was on human chromosome 6q25.2 between the* SCAF8* and* CNKSR* genes was found by Family Investigation of Nephropathy and Diabetes (FIND) using genome-wide association studies in multiple population [15]. Although we performed the association study in 1884 Chinese Han, we could not find obvious evidence of association between rs955333 and DKD in our sample. However, we found that this SNP was marginally associated with DR in both additive and recessive model. After we adjusted the confounding factor, it showed the subjects with GG genotype could be protected from diabetic retinopathy (*P* = 0.047). Our results also showed that subjects with GG had lower level of microalbuminuria than other genotypes. So we concluded that rs955333 was associated with diabetic retinopathy. Diabetic retinopathy is a microvascular complication of diabetes, just like DKD. Although clinical data shows DKD patients are always accompanied with diabetic retinopathy, not all the patients with diabetic retinopathy will develop DKD at last [20]. It suggests that both shared and complication-specific mechanisms contribute to the different microvascular disease phenotypes. Our result shows that the subjects with genotype GG on rs955333 have lower prevalence rate of diabetic retinopathy and lower level of microalbuminuria. We speculated genotype GG on rs955333 might influence the expression level of the genes on 6q25.2. Like* CNKSR3* which is an aldosterone receptor target gene and highly expressed in the renal cortical collecting duct and is upregulated in response to physiologic aldosterone concentrations to keep plasma volume [16], further research needed to test whether genotype GG on rs955333 could lower the expression of* CNKSR3* so as to lower the prevalence of diabetic retinopathy.

In this study, we did not repeat the same results as FIND, one possible reason might be that the statistical power of our sample was not enough to detect the effect of this locus, and although we had over 90% power to detect the association at the level based on previously reported ORs in non-Asian population (0.73), we could not exclude the possibility that the SNP might be less effective in Chinese population than other populations [21]. In this case, our sample may not have sufficient power. Secondly, the criteria we used to define DKD might be different from FIND, the subjects recruited by FIND (all had DM duration > 5 years and/or DR, with UACR > 1 g/g or ESKD) were more severe compared to us [15].

There are some limitations in our study. Firstly, all the subjects were located in shanghai or nearby regions; the population may have inherent bias. Second, subjects with advanced DKD should be recruited to further verify the genetic effect of the locus on DKD. Thirdly, the GWAS showed that the region 6q25.2 was associated with DKD, but we only tested one locus in our sample. On account of the population differences in the genetic architecture, we need to explore the region further.

## 5. Conclusion

Our data suggests rs955333 on chromosome 6q25.2 may not play a major role in DKD in Chinese population. However, we found evidence for association of rs955333 with diabetic retinopathy susceptibility in Chinese people with T2DM.

## Figures and Tables

**Table 1:** The clinical characteristics of DKD group and control group.

Characteristic	Control subjects	Diabetic kidney disease	*P* value
Male/female	719/570	326/209	**0.0427**
Age (years)	57.88 ± 9.81	60.14 ± 9.84	**0.0051**
BMI (kg/m^2^)	24.82 ± 3.53	25.02 ± 3.49	0.3047
Duration of diabetes (years)	10 (7, 14)	10 (6, 15)	0.8527
HbA1C (%)	8.75 ± 2.07	8.85 ± 2.11	0.3727
SBP (mmHg)	131.04 ± 16.28	135.45 ± 17.36	**<0.0001**
DBP (mmHg)	79.92 ± 9.21	81.31 ± 9.61	**0.0127**
MA (mg/24h)	10.85 (6.41, 25.17)	58.13 (33.79, 157.16)	**<0.0001**
eGFR (mL/kg/1.73 m^2^)	132.89 (115.95, 154.12)	101.59 (80.2, 132.25)	**<0.0001**
Uric acid (*μ*mol/L)	301.0 (255.0, 359.0)	346.0 (287.0, 413.0)	**<0.0001**
Creatinine (*μ*mol/L)	63 (53.37, 73.54)	80.12 (64.49, 101.29)	**<0.0001**
Blood urea nitrogen (mmol/L)	5.14 (4.23, 6.01)	6.04 (4.91, 7.53)	**<0.0001**
TC (mmol/L)	1.70 ± 1.4	2.11 ± 2.01	**0.0467**
TG (mmol/L)	4.70 ± 1.08	4.87 ± 1.37	**<0.0001**

Data are shown as the mean ± SD or median (interquartile range). The Wilcoxon test was used for the skewed distributed variables. *χ*^2^ test was used to determine proportions of the categorical variables. *P* values < 0.05 are shown in bold. DKD: diabetic kidney disease. BMI: body mass index. SP: systolic blood pressure. DP: diastolic blood pressure. MA: microalbuminuria. eGFR: estimated glomerular filtration rate. TC: total cholesterol. TG: total triglycerides.

**Table 2:** The clinical characteristics of diabetic retinopathy group and control group.

Characteristic	Control subjects	Diabetic retinopathy	*P* value
Male/female	628/492	319/226	0.3414
Age (years)	58.56 ± 9.87	57.76 ± 9.24	**0.0473**
BMI (kg/m^2^)	24.88 ± 3.53	24.84 ± 3.50	0.6125
Duration of diabetes (years)	9 (5, 12)	11 (8, 16)	**<0.0001**
HbA1C (%)	8.82 ± 2.1	8.80 ± 2.05	0.8048
SBP (mmHg)	130.67 ± 16.01	135.24 ± 17.68	**<0.0001**
DBP (mmHg)	80.35 ± 9.25	80.41 ± 9.29	0.8808
MA (mg/24h)	7.31 (3.46, 19.64)	8.34 (3.95, 26.33)	**0.0484**
eGFR (mL/kg/1.73 m^2^)	126.08 (106.75, 148.52)	128.75 (105.72, 151.72)	0.7612
Uric acid (*μ*mol/L)	309.5 (258.0, 369.0)	317.0 (269.0, 372)	0.1255
Creatinine (*μ*mol/L)	65.5 (54.0, 78.0)	66.0 (55.0, 78.0)	0.4402
Blood urea nitrogen (mmol/L)	5.3 (4.4, 6.3)	5.3(4.4, 6.4)	0.8090
TC (mmol/L)	1.84 ± 1.72	1.80 ± 1.72	0.8687
TG (mmol/L)	4.77 ± 1.15	4.76 ± 1.21	0.4843

Data are shown as the mean ± SD or median (interquartile range). The Wilcoxon test was used for the skewed distributed variables. *χ*^2^ test was used to determine proportions of the categorical variables. *P* values < 0.05 are shown in bold. DKD: diabetic kidney disease. BMI: body mass index. SP: systolic blood pressure. DP: diastolic blood pressure. MA: microalbuminuria. eGFR: estimated glomerular filtration rate. TC: total cholesterol. TG: total triglycerides.
